# Supplementary figures and images for: Prevalence and antimicrobial resistance pattern of Clostridium difficile among hospitalized diarrheal patients: A systematic review and meta-analysis
Source: PLoS One. 2022 Jan 13;17(1):e0262597. doi: 10.1371/journal.pone.0262597 (PMC8758073; doi:10.1371/journal.pone.0262597)

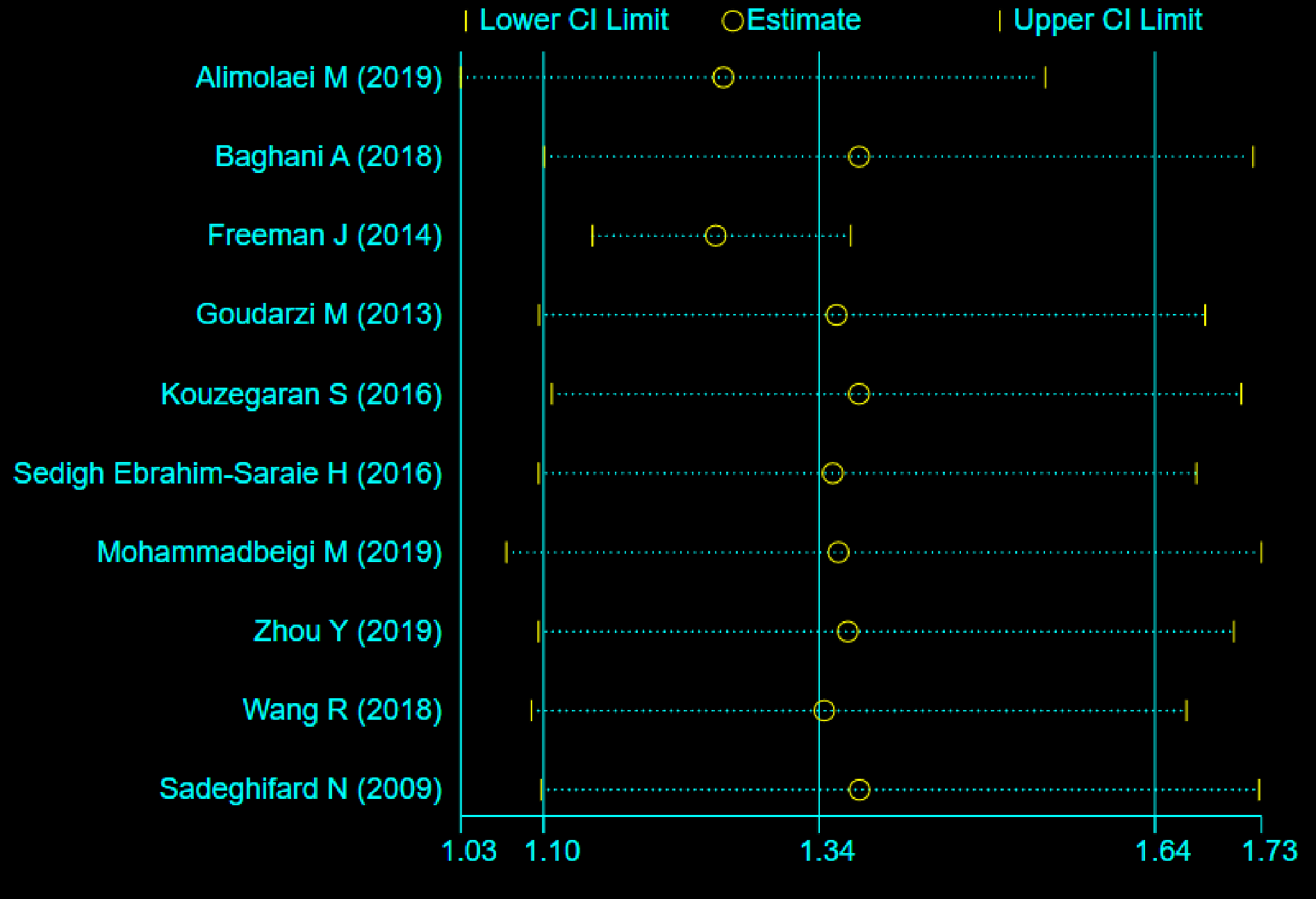

Supplement: S1 Fig — (TIF) [file pone.0262597.s002.tif]

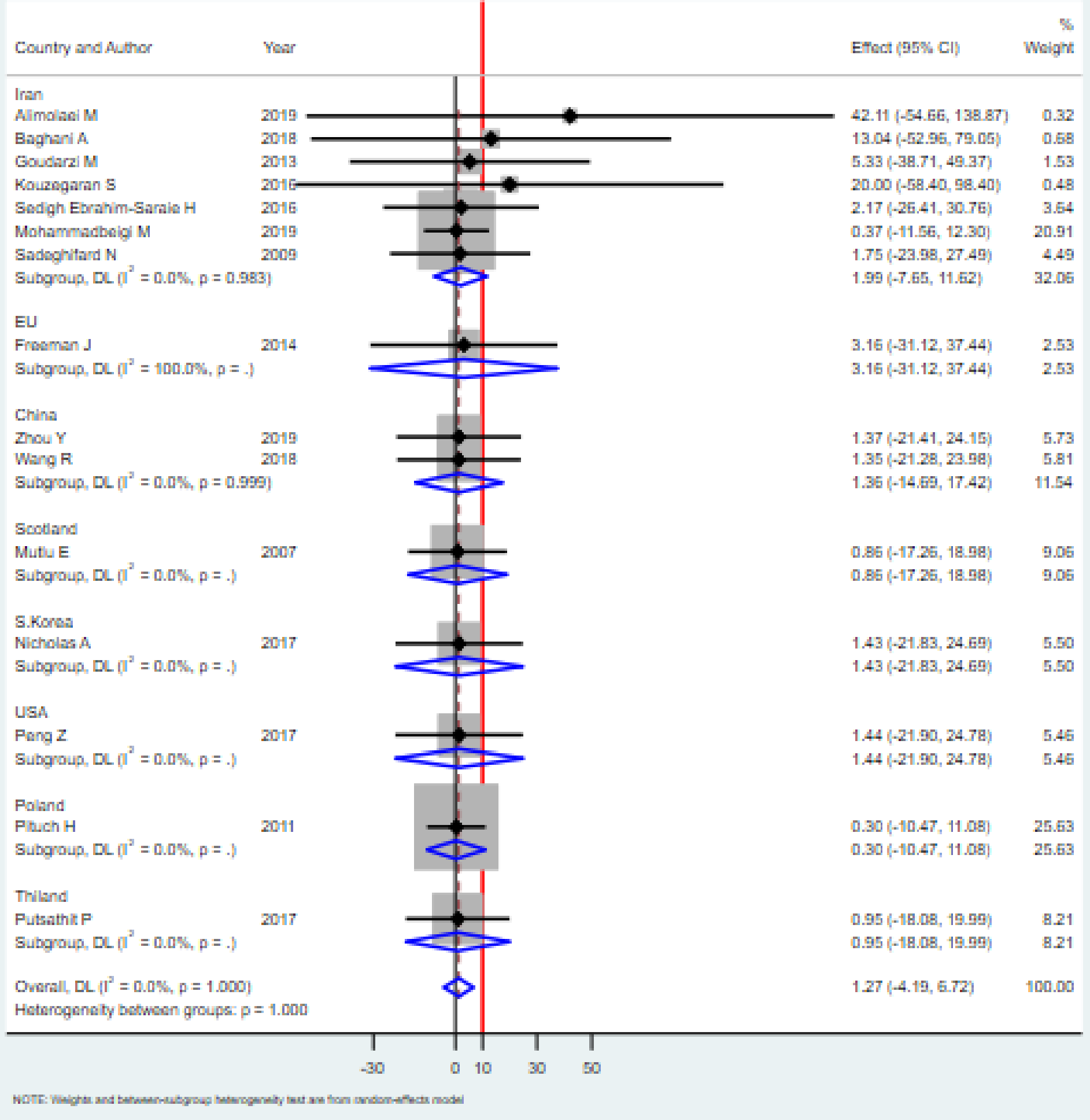

Supplement: S2 Fig — (TIF) [file pone.0262597.s003.tif]

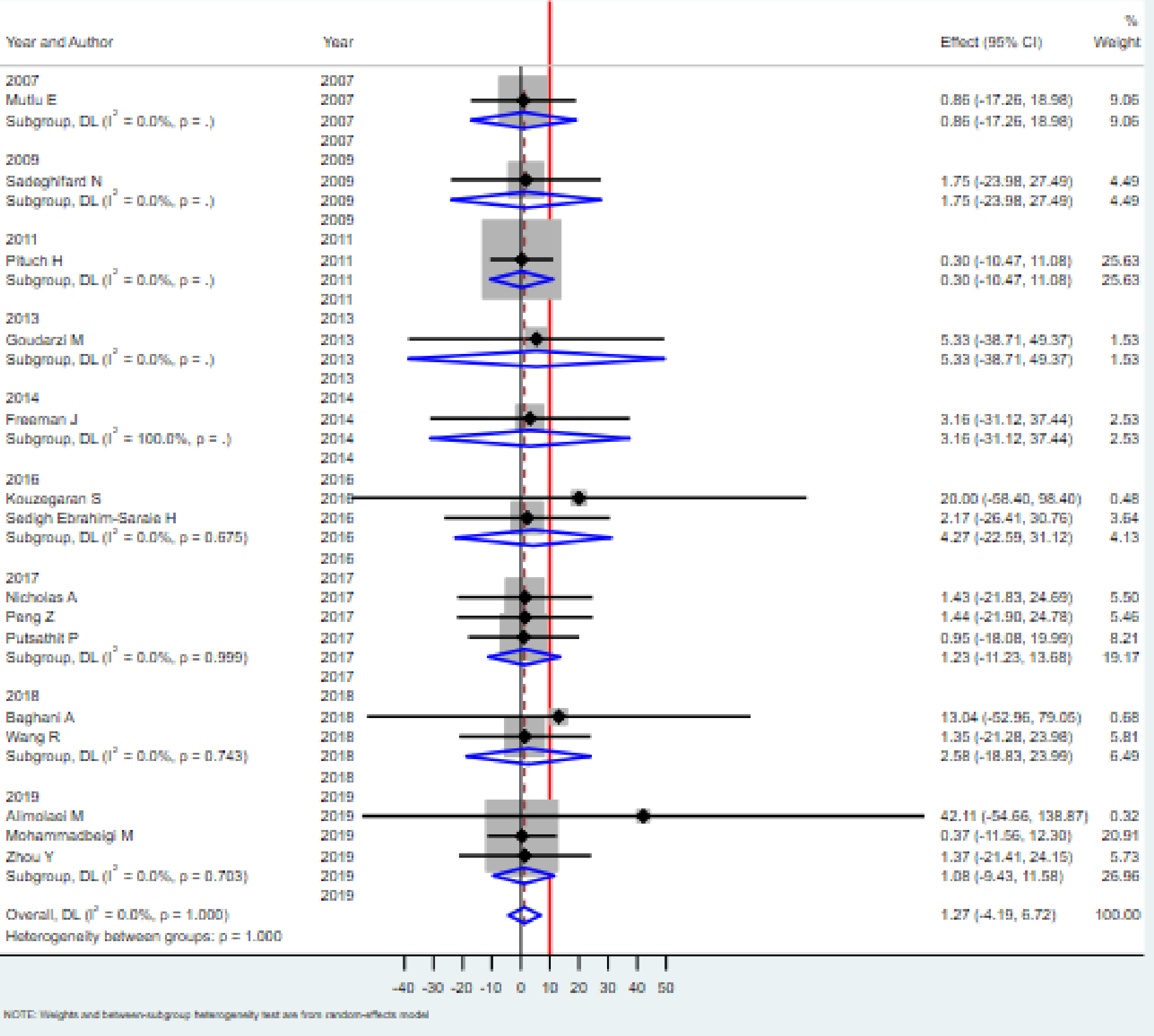

Supplement: S3 Fig — (TIF) [file pone.0262597.s004.tif]

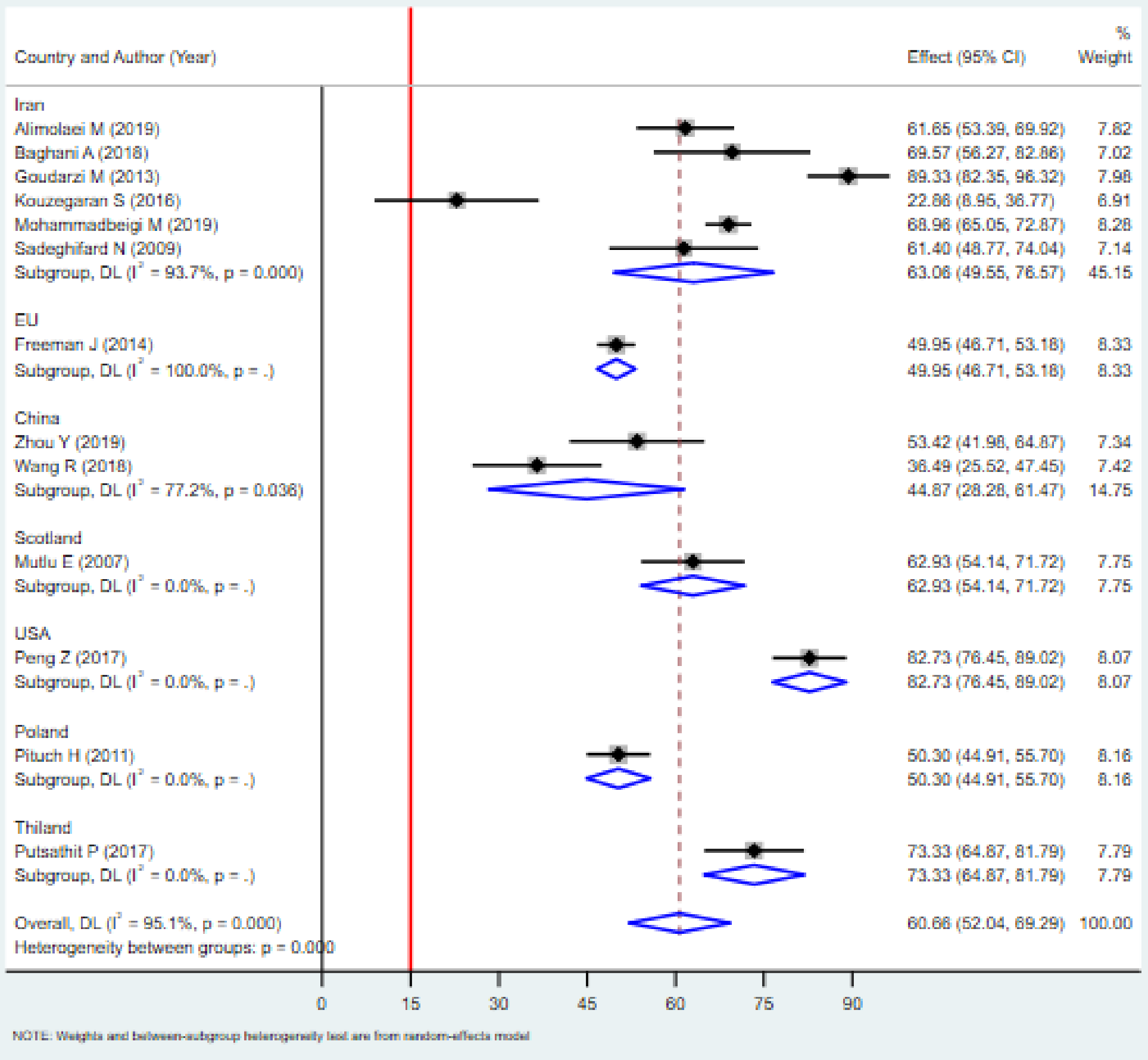

Supplement: S4 Fig — (TIF) [file pone.0262597.s005.tif]
