# Supplementary material for: Prevalence and antimicrobial resistance pattern of Clostridium difficile among hospitalized diarrheal patients: A systematic review and meta-analysis
Source: PLoS One. 2022 Jan 13;17(1):e0262597. doi: 10.1371/journal.pone.0262597 (PMC8758073; doi:10.1371/journal.pone.0262597)
Supplement: Attachment — Submitted filename: Response to Reviewers.doc [file pone.0262597.s006.doc]

**Response letter**

**Dear Dr. Yung-Fu Chang**

**Academic Editor, PLoS One**

This is a revised manuscript (PONE-D-21-16139R1) entitled: “**Prevalence and antimicrobial resistance pattern of *Clostridium difficile* among hospitalized diarrheal patients: a systematic review and meta-analysis**” to be published in **PLoS One**.

By: Tebelay Dilnessa (Assis. Professor)^*^, Alem Getaneh (MSc), Workagegnehu Hailu (Medical Doctor, Internist), Feleke Moges (Professor) and Baye Gelaw (Professor)

**Dear Academic Editor,**

First, once again we would to acknowledge you for comments forwarded which is very important for the improvement of the manuscript. We would like to thank very much the reviewer for providing important comments for our manuscript. Then, please find the attached revised documents that were accommodated with the comments forwarded by the academic editor and reviewer.

**Responses to Academic Editor:**

**Academic Editor**: Journal Requirements: Please review your reference list to ensure that it is complete and correct. If you have cited papers that have been retracted, please include the rationale for doing so in the manuscript text, or remove these references and replace them with relevant current references. Any changes to the reference list should be mentioned in the rebuttal letter that accompanies your revised manuscript. If you need to cite a retracted article, indicate the article’s retracted status in the References list and also include a citation and full reference for the retraction notice.

**Author response**: Thank you for sharing the PLOS ONE's journal requirements for references. We went through each reference and checked online on databases its availability. We got all references used in the manuscript were complete, correct and available in the databases. There are no retracted references used in this manuscript. Again, we made no any changes to the references list.

**Responses to Reviewer #1:**

Thank you for your comments and we accepted comments and incorporated to the manuscript.

**Reviewer 1**: The language needs to be checked and edited again. In line 370, the word "was" is corrected to "were". In line 371, The pooled resistances...In line 376, no resistant strains have been identified.... e.g.

**Author response**: Thank you for the comment. A comprehensive English language editing, revisions and topographical corrections were made throughout the manuscript. For example:-

- Metronidazole and vancomycin were still the drugs of choice…. (line 370)
- The pooled resistances of *C. difficile* against…….(line 371)
- No resistant strains have been identified…… (376)
- Further, erythromycin and tetracycline resistances were observed……. (384),
- Etc, were revised and corrected.

Finally, once again we would like to thank academic editor and the reviewer.

In case of any questions and doubts, please do not hesitate to contact us anytime.

Tebelay Dilnessa
